# Using Multi-Modal Electronic Health Record Data for the Development and Validation of Risk Prediction Models for Long COVID Using the Super Learner Algorithm

**DOI:** 10.3390/jcm12237313

**Published:** 2023-11-25

**Authors:** Weijia Jin, Wei Hao, Xu Shi, Lars G. Fritsche, Maxwell Salvatore, Andrew J. Admon, Christopher R. Friese, Bhramar Mukherjee

**Affiliations:** 1 Department of Biostatistics, School of Public Health, University of Michigan, Ann Arbor, MI 48109, USA; wjjin@umich.edu (W.J.);; 2 Center for Precision Health Data Science, School of Public Health, University of Michigan, Ann Arbor, MI 48109, USA; 3 Department of Epidemiology, University of Michigan, Ann Arbor, MI 48109, USA; 4 Department of Internal Medicine, Division of Pulmonary and Critical Care Medicine, School of Public Health, University of Michigan, Ann Arbor, MI 48109, USA; 5 VA Center for Clinical Management Research, Ann Arbor, MI 48109, USA; 6 LTC Charles S. Kettles VA Medical Center, Ann Arbor, MI 48109, USA; 7 School of Nursing, University of Michigan, Ann Arbor, MI 48109, USA; 8 Institute for Healthcare Policy and Innovation, School of Public Health, University of Michigan, Ann Arbor, MI 48109, USA

**Keywords:** COVID-19, post-acute sequelae of SARS-CoV-2 infection, electronic health records, predictive models, phenotype risk score

## Abstract

Background: Post-Acute Sequelae of COVID-19 (PASC) have emerged as a global public health and healthcare challenge. This study aimed to uncover predictive factors for PASC from multi-modal data to develop a predictive model for PASC diagnoses. Methods: We analyzed electronic health records from 92,301 COVID-19 patients, covering medical phenotypes, medications, and lab results. We used a Super Learner-based prediction approach to identify predictive factors. We integrated the model outputs into individual and composite risk scores and evaluated their predictive performance. Results: Our analysis identified several factors predictive of diagnoses of PASC, including being overweight/obese and the use of HMG CoA reductase inhibitors prior to COVID-19 infection, and respiratory system symptoms during COVID-19 infection. We developed a composite risk score with a moderate discriminatory ability for PASC (covariate-adjusted AUC (95% confidence interval): 0.66 (0.63, 0.69)) by combining the risk scores based on phenotype and medication records. The combined risk score could identify 10% of individuals with a 2.2-fold increased risk for PASC. Conclusions: We identified several factors predictive of diagnoses of PASC and integrated the information into a composite risk score for PASC prediction, which could contribute to the identification of individuals at higher risk for PASC and inform preventive efforts.

## 1. Introduction

Despite the declaration by the WHO on 5 May 2023 marking the end of the COVID-19 emergency [1], the long-term clinical consequences of COVID-19 continue to pose significant health challenges [2,3,4]. Post-Acute Sequelae of COVID-19 (PASC), also known as Post-COVID Conditions (PCC) [5], Long COVID [6], Post-Acute COVID-19 Syndrome (PACS) [7], and Long Haul COVID-19 [8], encompass a diverse array of persistent symptoms and new chronic disorders that can arise following a COVID-19 infection. These can range from lingering symptoms following the initial infection, such as cough, fatigue, and loss of smell [9,10,11], to the development of chronic lung or neurological conditions [4,12,13,14,15,16,17], as well as late post-COVID complications, including autoimmune complications. The global prevalence of PASC is estimated to be substantial, with at least 65 million individuals worldwide affected [18]. Advancing our knowledge of the physiological mechanisms underlying PASC plays a crucial role in accurately predicting PASC and enabling early identification of patients at high risk [19], who may then be candidates for PASC-focused treatment or follow-up care [9,20].

Clinically defined as the continuation or development of new symptoms 3 months after the initial SARS-CoV-2 infection, PASC have been associated with a wide range of factors, including demographic characteristics such as female gender [21], older age [22], and higher BMI [23,24], as well as pre-existing conditions (e.g., fatigue, respiratory system disorders [11,25]). Additionally, certain medication use (e.g., angiotensin-converting enzyme inhibitors and metformin), genetic factors (e.g., FOXP4 locus), and environmental factors (e.g., engagement in transportation, logistics, or the discipline workforce) have been revealed to be predictive of PASC diagnosis [26,27,28,29]. Efforts have also been made to predict PASC based on these factors using various methods, including flexible machine learning models such as XGBoost [30], random forest [31,32], deep neural network [33], and logistic regression models [34,35]. These studies highlight the importance and feasibility of identifying risk factors related to PASC and predicting patients with a high risk of developing PASC. However, most of these studies have relied on specific risk factors (e.g., genetics or pre-existing comorbidities) rather than modeling them jointly. There is also limited literature comparing the relative contribution of each data domain to the prediction of PASC that can inform future studies employing primary data collection. Therefore, there is a need to integrate information from multi-modal data and develop integrated predictive tools to enhance our understanding and predictions of PASC.

To address this research gap, we used electronic health record (EHR) data from a comprehensive cohort of 92,301 COVID-19-positive patients who received care at Michigan Medicine (MM), a large academic medical center in the midwestern United States, from March 2020 to January 2023. Leveraging this EHR data, we developed prediction models using time-referenced clinical phenotype data, medications, and laboratory measurements during the pre-infection and acute infection periods of COVID-19. Recognizing the heterogeneity among patient populations and feature spaces, one single prediction model might not perform well in each of the data domains, so we constructed the prediction models using the Super Learner, an ensemble predictive algorithm [36]. Introduced in 2007, Super Learner uses cross-validation to arrive at the optimal weighted combination of base learners. Super Learner demonstrated superior performance compared to individual machine learning algorithms [37], which was also observed in predictions related to COVID-19 or Long COVID [38]. Through this exercise, we identified important features that are predictive of PASC in a training dataset based on their permutation importance [39]. We then constructed five integrated risk scores (RSs) using different data domains and time periods. These risk scores, when combined, show promising prediction and risk stratification performance in a test dataset. With new diseases like COVID-19, which emerged three years ago, in the absence of long-term population-based studies, one often must rely on the EHRs for clues contributing to an emerging etiology of the disease and its sequelae. Our study offers a principled framework to integrate multiple data domains to quantify their joint and individual contributions to the prediction of PASC and, thus, enhance our understanding of this complex and heterogeneous condition.

## 2. Materials and Methods

### 2.1. Study Cohort

The study cohort consisted of 92,301 eligible individuals who were patients at Michigan Medicine (MM) and had a recorded COVID-19 diagnosis or a positive reverse transcriptase polymerase chain reaction (RT-PCR) test for a severe acute respiratory syndrome coronavirus 2 (SARS-CoV-2) infection performed or recorded at MM between 10 March 2020 and 3 January 2023. Only the first SARS-CoV-2 infection was recorded.

We categorized the COVID-19-positive patients into two groups: those with a recorded diagnosis of PASC and those without any recorded PASC diagnosis, referred to as the “no PASC” group. Consistent with our previous work [35], we employed the same definition for PASC diagnoses: a PASC record in the Problem Summary List (PSL) or a U09.9 (“Post COVID-19 condition, unspecified”) or B94.8 (“Sequelae of other specified infectious and parasitic diseases”) ICD-10-CM diagnosis code.

### 2.2. Covariate Definition

To examine and adjust for confounding by patient characteristics, socioeconomic status, and other variables, we also collected the following data for each participant: age, self-reported gender, self-reported race/ethnicity, Neighborhood Disadvantage Index (NDI) without proportion of Black (coded as quartiles, with larger quartiles representing more disadvantaged communities) [40,41], population density measured in persons per square mile (operationalized as quartiles), vaccination status, body mass index (BMI, the last measurement before the index date (represents the date of the first positive COVID-19 test or diagnosis (day 0))), Elixhauser comorbidity score [42,43], COVID-19 severity (non-severe (not hospitalized) and severe (hospitalized or deceased)), healthcare worker (HCW) status (yes or no), and time span of records in the EHR before the COVID-19 test. These time spans were based on the first or last recorded encounter in the EHR data.

### 2.3. Matching

**Figure 1:**
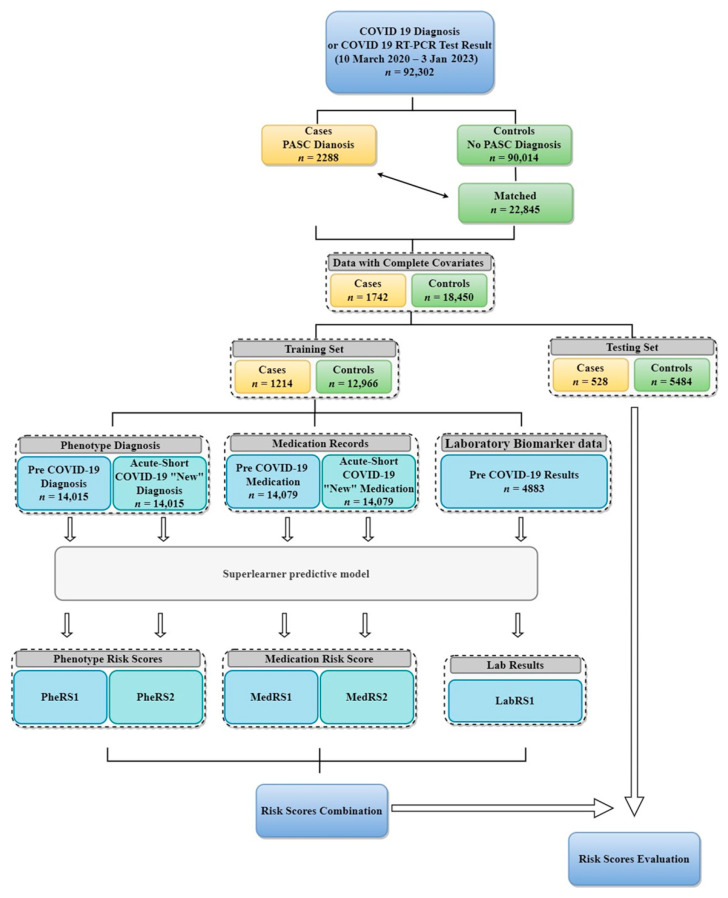
Overview flowchart showing the sample filtering and analysis setup.

The flow diagram describing the analytic steps undertaken in our study is presented in Figure 1. First, to minimize potential bias introduced by the covariates to the exploration of predictive multi-modal EHR data and improve the comparability between PASC and “No PASC” COVID-19 patients, we matched each PASC COVID-19 patient (referred to as cases) with up to 10 “No PASC” COVID-19 patients (referred to as controls) using the “MatchIt” R package [44]. Nearest neighbor matching was employed for age at index date, pre-COVID-19 years in the EHR, and post-COVID-19 years in the EHR. Exact matching was applied for sex, primary care visit at Michigan Medicine within the last 2 years (yes/no), race/ethnicity, and year quarter of the index date. After matching, we randomly split the matched patients into training and testing sets with a ratio of 7:3 to create and evaluate the performance of the predictive model.

### 2.4. Reference Time Period

As depicted in Figure S1, our analysis considers two distinct time periods based on the index date. The pre-COVID-19 period included the time period from 2 years to 14 days prior to the index date (−2 years to −14 days), while the acute COVID-19 period covered the period from 14 days before the index date to 28 days after the index date (−14 days to +28 days). Accordingly, we partitioned the EHR contents into these two distinct time periods to capture associations specific to the pre-COVID-19 and acute COVID-19 periods.

### 2.5. Processing of Multi-Modal Data

In addition to the covariates mentioned earlier, our dataset includes multi-modal data encompassing three primary domains: medical phenotype data (signs, symptoms, and diagnoses), medication records, and laboratory biomarker measurements.

#### 2.5.1. Phenotype Data

To construct each subject’s medical phenome, we extracted the available International Classification of Diseases (ICD; ninth and tenth editions [45,46]) codes from the EHR during the two defined time periods. These codes were then mapped to 1813 phenotype concepts known as PheCodes using the R package “PheWAS” [47], separately. In short, each patient was assigned a binary value of “1” if they had ICD codes corresponding to a specific PheCode during the respective period, and “0” if not. This process generated two time period-specific phenomes for each patient: the pre-COVID-19 period phenome and the acute COVID-19 period phenome. To further distinguish between the two time periods, we created an additional phenome that captured only diagnosed conditions that exclusively appeared during the acute COVID-19 period and did not appear in the pre-COVID phenome for each patient. Furthermore, to avoid incorporating sparse records and to prevent the inclusion of records that are present in only males or females, we excluded PheCodes with fewer than 10 occurrences in either gender (male or female). As a result, we obtained two phenomes for further analysis: the “pre-COVID-19 period phenome” (19,956 patients and 1508 PheCodes) and the “acute-COVID-19 period new phenome” (19,956 patients and 526 new PheCodes).

#### 2.5.2. Medication Data

Similarly, to construct each subject’s medication history, or “medicome”, we extracted the available list of medication order and administration data from the EHR and mapped them to Anatomical Therapeutic Chemical (ATC) codes. Specifically, we focused on the fourth level of the ATC codes, as it provides more specific and clinically relevant information while avoiding redundancy, and yielded 942 ATC codes. Following the same principle as the phenome data, we divided the medicome into two distinct periods and generated a new medicome that included newly used medications that exclusively appeared during the acute COVID-19 period. After excluding ATC codes with fewer than 10 occurrences in either gender, we obtained two medicomes: “pre-COVID-19 medicome” (20,040 patients and 409 ATC codes) and “acute-COVID-19 new medicome” (20,040 patients and 276 new ATC codes).

#### 2.5.3. Laboratory Biomarker Data

We also retrieved the clinical laboratory measurements corresponding to each patient from their respective EHRs. We focused on 42 specific laboratory traits that have been commonly analyzed for their association with COVID-19 prognosis (Table S1). With repeated laboratory tests, the median value of each individual’s laboratory measurements during the specific time period was considered as their summary laboratory measure. Due to the high degree of missingness of the laboratory biomarker during the relatively short acute COVID-19 period, only laboratory measurements from the pre-COVID-19 period were included in our analysis. To maximize sample size, we utilized “univariate regression models” as a basic screening tool for laboratory measures. In this approach, a logistic regression model (Equation (1)) was individually applied to each laboratory measure to capture their adjusted correlations with PASC (as indicated by the *p*-value of the coefficient).

(1) $$\begin{array}{c} logit\left(P\left(PASC\;is\;present|Covariates,\;Laboratory\;Measurements\right)\right)\\ =\beta_{0}+\sum_{p=1}^{P}\beta_{p}Covariate_{p}+\beta_{Lab}Lab \end{array}$$

where *P* represents the number of covariates.

The top 15 Laboratory Result Codes with the lowest *p*-values were selected and included in the “pre COVID-19 period lab” data (6987 patients and 15 Laboratory Result Codes).

Consequently, we obtained five distinct data domains: “pre-COVID-19 phenome”, “acute-COVID-19 new phenome”, “pre COVID-19 medicome”, “acute-COVID-19 new medicome”, and “pre- COVID-19 laboratory measurements”.

### 2.6. Statistical Methods for Feature Identification and Risk Score Construction

We constructed a Super Learner (SL)-based predictor to generate an integrated risk score using each of the five data domains in addition to the set of basic covariates [36]. The Super Learner algorithm is an ensemble machine learning algorithm that uses V-fold cross-validation to build the optimal weighted combination of predictions from a library of candidate algorithms. Specifically, each data domain was first evaluated separately by using a 10-fold cross-validated SL trained on the training set using PASC status as a binary outcome (Equation (2)).

(2) $${\hat{y}}_{SL,D_{j}}=\hat{P}\left(PASC\;is\;present|Covariates,D_{j}\right)=f_{SL}\left(Covariates,D_{j}\right)$$

where j=1,2,…,5, $D_{j}$, represents the *j*-th data domain (pre- and acute COVID phenome and medicome, and pre-COVID labs). The SL model incorporated five types of learners, including random forest, Generalized Linear Model, elastic net, XGBoost, and Bagging Classification Trees. Subsequently, a risk score (RS) was calculated for each patient using the predicted logit function of PASC (Equation (3)).

(3) $$RS(D_{j})=logit\left({\hat{y}}_{SL,D_{j}}\right)$$

Subsequently, we calculated five distinct risk scores based on the five data domains to capture the respective risk factors associated with PASC. These risk scores included phenotype risk scores 1 and 2 (PheRSs 1 and 2), which summarized the pre-COVID and acute COVID phenotype-related risk factors, medication risk scores 1 and 2 (MedRSs 1 and 2), which summarized the pre-COVID and acute COVID medication-related risk factors, and laboratory risk score 1 (LabRS1), which summarized the pre-COVID laboratory-related risk factors.

In addition to the risk scores, we evaluated the feature importance to gain a comprehensive understanding of the contribution of different risk factors arising from each data domain. Permutation importance was used for this purpose using the vip package [39,48], which measures the decrease in model AUC (area under the ROC curve) when the corresponding feature is randomly shuffled. To ensure stability, each importance was estimated using 10 Monte Carlo replications. For validation purposes, we also calculated the SHAP-based variable importance score of the features using the vip package.

### 2.7. Risk Score Combination

To combine the information captured by different periods and evaluate the prediction contribution of each data domain series (e.g., phenome, medicome), we fit a ridge-penalized logistic regression on the training set and obtained the estimated weight corresponding to each RS (Equation (4)). The weights estimated for each risk score when combined are provided in Table S3.

(4) $$\begin{array}{c} logit\left(y|Covariates,RSs\right)=logit\left(P\left(PASC\;is\;present|Covariates,\;RSs\right)\right)\\ =\beta_{0}+\sum_{p=1}^{P}\beta_{p}Covariate_{p}+\sum_{k=1}^{K}\beta_{RS_{k}}RS_{k} \end{array}$$

where $\hat{\beta^{RIDG}}=\left({\hat{\beta }}_{0},{\hat{\beta }}_{1},{\hat{\beta }}_{2},\ldots ,{\hat{\beta }}_{P},{\hat{\beta }}_{RS_{1}},{\hat{\beta }}_{RS_{2}},\ldots ,{\hat{\beta }}_{RS_{K}}\right)=\underset{\beta }{arg\;min}\left\{-\sum_{i=1}^{N}\left[y_{i}log\left(\pi_{i}\right)+\left(1-y_{i}\right)\log(1-\pi_{i})\right]+\lambda ||\beta |{|}_{2}^{2}\right\}$ represents the ridge-penalized estimator for the coefficients. *P* denotes the number of covariates, *K* denotes the number of RSs to be combined, $\pi_{i}$ is the predicted probability of $y_{i}=1$ of *i*-th individual based on β, and *N* denotes the number of individuals used for training.

The combined risk scores were then calculated as the weighted sum of a set of selected RSs (Equation (5)).

(5) $$RS_{combined}=\sum_{k=1}^{K}{\hat{\beta }}_{RS_{k}}RS_{k}$$

We constructed a phenotype risk score (PheRS) using a weighted combination of PheRS1 and PheRS2, and a medication risk score (MedRS) using a weighted combination of MedRS1 and MedRS2. Afterwards, to further combine the information captured by different risk scores, we combined PheRS, MedRS, and LabRS1 to create a composite risk score, “AllRS”.

### 2.8. Risk Scores Evaluation

To evaluate each of the RSs we generated (e.g., PheRS1, MedRS1, PheRS), we fitted a Firth bias-corrected logistic regression model for each RS, adjusting for age, gender, race/ethnicity, Elixhauser score, population density, NDI, HCW, vaccination status, BMI, pre-COVID-19 years in EHR, and COVID19 severity, using a complete case analysis.

We then assessed the following performance measures relative to the PASC status on the testing set: (1) overall performance of the risk score as measured by the Nagelkerke’s Pseudo-R^2^ of the model using the R package “rcompanion”; (2) accuracy with the Brier score using the R package “DescTools”; (3) ability to discriminate between PASC cases and matched controls as measured by the area under the covariate-adjusted receiver operating characteristic (AROC; semiparametric frequentist inference) curve (denoted AAUC) using the R package “ROCnReg”; (4) and (5) overall association with PASC as measured by the odds ratio (OR) and *p*-value of the predictor corresponding to PASC when adjusting for the covariates. To compare effect sizes corresponding to the various predictors, we have centered each predictor to their mean and scaled them to have a standard deviation of 1 during this analysis.

Finally, we conducted a risk stratification analysis using an aggregate score derived from a select set of predictors. We partitioned the control group’s aggregate score distribution into deciles to establish our risk categories based on the training set. Then, within the testing set, we allocated the PASC cases into these deciles, thus allowing us to profile disease prevalence across the different risk categories. To further demonstrate the risk stratification ability, we calculated the OR of PASC corresponding to an RS higher than its 90th percentile in the training set. This was achieved by fitting covariates-adjusted logistic regression models in the testing data using different composite risk scores. Furthermore, the odds ratio (OR) for each decile was also calculated, using the middle of the risk score (40–60th percentile) as the reference level.

## 3. Results

### 3.1. Patient Characteristics

Among the 92,301 COVID-19-positive patients who were seen in MM at least two months after their first COVID-19 diagnosis or positive RT-PCR test, a total of 2287 (2.5%) received a diagnosis of PASC. Analysis revealed notable differences in patient characteristics between PASC cases and controls. PASC cases, on average, were older at their index date, with a mean age of 47.93 years compared to 42.29 years for the controls (Table 1). Moreover, a higher proportion of females was observed among PASC cases, accounting for 65.0% of PASC cases compared to 57.3% among the controls. Additionally, the proportion of patients receiving primary care at MM was significantly higher in PASC cases (58.1%) compared to PASC controls (45.3%). To address these observed differences and mitigate potential bias, matching was performed on several variables (see Section 2). All significant differences in covariates became nonsignificant after matching (Table 1).

Furthermore, apart from the matching variables, several other variables exhibited different distributions between matched cases and controls. For instance, matched cases had higher average Elixhauser scores (4.67 versus 3.94), lower rates of booster vaccination (8.7% versus 10.5%), and a lower proportion of healthcare workers (2.8% versus 3.5%). These univariate summary findings suggested that these variables distribute quite differently among PASC cases and controls conditioned by the matching process. Combined with the results from previous studies [20,35], it is plausible that these variables could serve as potential predictive factors for PASC, and we included them in the prediction models.

### 3.2. Key Predictive Features Identified by the SL Algorithm in Training Data

#### 3.2.1. Predictive Phenotypes

**Figure 2:**
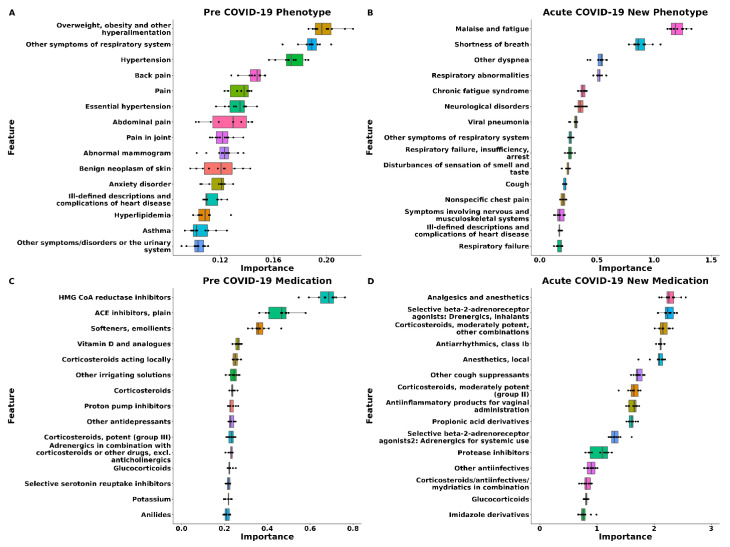
Feature importance plots for phenotype and medication data on the training set. All importance has been multiplied by 100. Importance was evaluated by the permutation importance (defined as the decrease in model AUC after the corresponding feature was randomly shuffled) based on the Super Learner model with the covariates being adjusted. Each importance was estimated by 10 Monte Carlo replications. The importance of all the covariates can be found in Table S2. Only 15 features with highest importance were presented. (**A**): Feature importance plot for phenotypes in the Super Learner (SL) model with pre-COVID phenotypes; (**B**): feature importance plot for phenotypes in the SL model with acute COVID new phenotypes; (**C**): feature importance plot for medications in the SL model with pre-COVID medications; (**D**): feature importance plot for medications in the SL model with acute COVID new medications.

Having investigated the demographic characteristics of PASC cases and controls, we now turn our attention to the phenotype factors that were predictive of the diagnosis of PASC. Figure 2 presents the top 15 most important features (as determined by permutation importance) when constructing the SL models. We also present the SHAP-based variable importance scores in Figure S2. In the models utilizing phenotype data during the pre-COVID period, overweight, obesity, and circulatory system signs and symptoms including hypertension were identified as diagnoses predictive of PASC. This aligns with high SHAP importance scores for overweight and hypertension. During the acute COVID period, predictive signs and symptoms mainly revolved around the respiratory system, including shortness of breath and respiratory abnormalities. Additionally, symptoms such as malaise and fatigue and chronic fatigue syndrome made significant contributions to the prediction of PASC (permutation importance of 0.012 and 0.0035). Additionally, malaise, fatigue, and chronic fatigue syndrome significantly contributed to PASC prediction (permutation importance of 0.012 and 0.0035), as consistently observed, illustrated in Figure S2. Notably, among the 10 permutations, the importance of features during the pre-COVID-19 period showed greater variability and smaller importance values compared to the acute COVID-19 period, implying that despite smaller time periods and fewer occurrences, diagnoses in the acute COVID-19 period have a stronger and more stable contribution to prediction.

#### 3.2.2. Predictive Medications

Moving on to the predictive medications as extracted from the medication administration and order records, the use of HMG CoA reductase inhibitors and ACE inhibitors exhibited the highest predictive importance among pre-COVID-19 medications. While there is a slight lack of overlap in the top 15 predictive features between the two lists, the importance of HMG CoA reductase inhibitors and ACE inhibitors is still evident (Figure 2 and Figure S2). Among acute COVID medications, analgesics, anesthetics, and selective beta-2-adrenoreceptor agonists were identified as the most predictive, supported by both permutation importance and SHAP-based scores. Among acute COVID medications, analgesics, anesthetics, and selective beta-2-adrenoreceptor agonists were identified as the most predictive. Furthermore, cough suppressants were also found to be predictive, which aligns with the well-known association between cough and both COVID-19 and Long COVID, as well as our results on the relationship between cough and Long COVID. Again, we also observed a higher importance in the acute COVID-19 period than the pre-COVID-19 period.

#### 3.2.3. Predictive Laboratory Measurements and Covariates

We also explored the predictive lab measurements during the pre-COVID period. Table S2 presents the screening results for the 42 lab results based on a complete analysis. Among them, hematocrit level, hemoglobin level, and red blood cell count emerged as the top three laboratory measurements associated with PASC and were all positively associated with a higher risk of PASC. However, we found that the association between most lab measurements and PASC was relatively weak, as only five lab measurements reached nominal significance (*p* < 0.05). Given our main objective of predicting PASC rather than solely identifying statistically significant predictors, and to maintain an adequate sample size, we expanded our analysis to include the top 15 significant hits and performed an SL model. The results, shown in Figure S3, indicate several lab measurements with similar importance, including segmented neutrophils, red cell distribution width, and hemoglobin.

Apart from the five data domains extracted from the EHR, we are also interested in the predictive performance of the covariates. Although matching has been applied to several covariates, our analysis indicates that among unmatched covariates, BMI, COVID-19 vaccination status, and COVID-19 severity (hospitalization) consistently displayed high importance across the six models presented. This consistent pattern suggests that these covariates possess predictive relevance for PASC. These findings align with previous studies highlighting the importance of these factors in the PASC risk assessment [24,49,50,51,52].

### 3.3. Risk Score Evaluation in Testing Data

After investigating the predictive risk factors in the SL model, we proceeded to assess the predictive power of the constructed risk scores.

#### 3.3.1. Distinct Risk Scores

**Figure 3:**
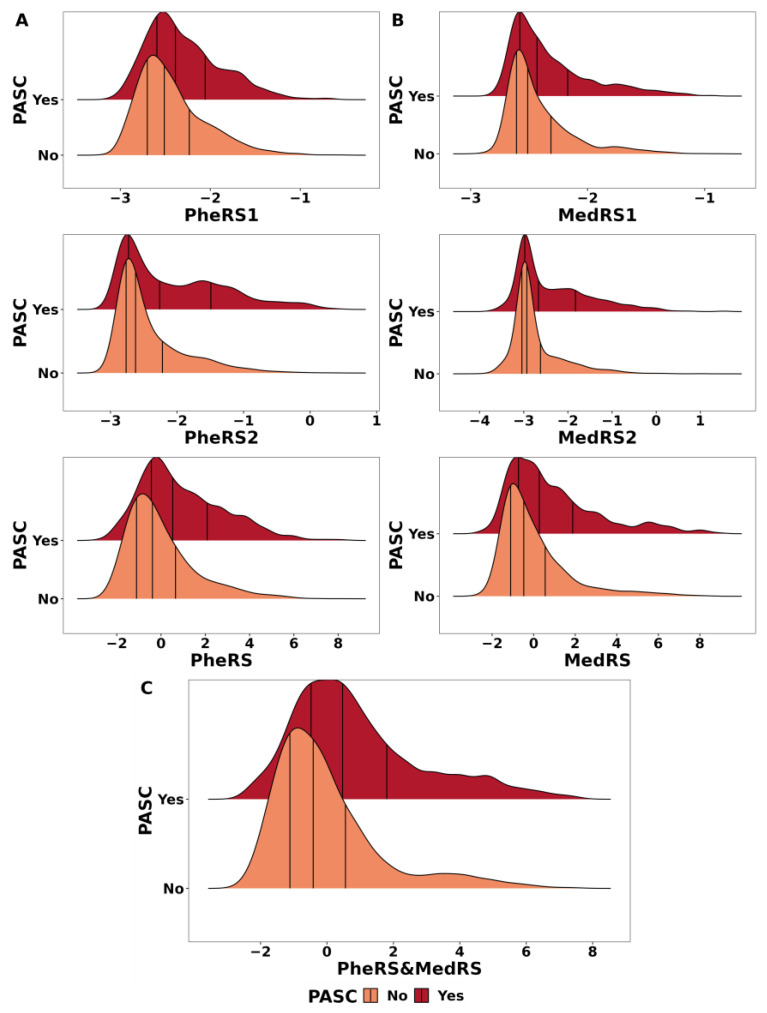
Risk score distribution among cases and controls in the testing set. (**A**): Distribution of phenotype risk scores (PheRS1: pre-COVID risk score; PheRS2: “Post COVID New Diagnosis” risk score; PheRS: combination of PheRS1 and PheRS2) in the testing set (*n* cases: 456, *n* controls: 3610); (**B**): distribution of medication risk scores (MedRS1: pre-COVID risk score; MedRS2: “New Medication” risk score; MedRS: combination of MedRS1 and MedRS2) on the testing set (*n* cases: 525, *n* controls: 5436); (**C**): distribution of PheRS and MedRS (combination of PheRS and MedRS) in the testing set (*n* cases: 454, *n* controls: 3603).

Firstly, focusing on the phenotype risk scores, we analyzed a testing set comprising 456 PASC cases and 3610 controls. Both PheRS1 and PheRS2 demonstrated a moderate discrimination ability for PASC, with AAUC values of 0.56 (0.53, 0.58) and 0.64 (0.61, 0.67), respectively (Table 2). Notably, PheRS2 exhibited a better model performance (Pseudo-R^2^ (PheRS2): 0.095 versus Pseudo-R^2^(PheRS1): 0.033) and a stronger PASC risk stratification ability (*p*-value (PheRS2): 2.97 × 10^−38^ versus *p*-value (PheRS1): 4.88 × 10^−8^). This suggests that the phenotype risk score derived from the short-acute COVID-19 period contains more information related to PASC than the pre-COVID period. The combination of these two phenotype risk scores, denoted as PheRS, yielded a slightly stronger discrimination ability, with an AAUC of 0.65 (0.62, 0.67), and a stronger risk stratification ability (adjusted OR (2.96 (2.49, 3.5), versus 1.94 (1.75, 2.14) and 1.55 (1.33, 1.82)). This improvement is further supported by Figure 3A, which visually illustrates a distinct right shift in the distribution of PheRS between PASC cases and controls compared to the individual risk scores. The concentration of PheRS in PASC cases implies that it effectively captures the risk factors associated with PASC, making it a valuable component in our prediction model.

Similar findings were observed for the medication risk scores. MedRS2 demonstrated better performance in predicting PASC compared to MedRS1 (AAUC(MedRS1): 0.6 (0.58, 0.63) versus AAUC(MedRS2): 0.53 (0.50, 0.56)). Combining these two risk scores resulted in a slight improvement in risk stratification ability as indicated by a higher adjusted OR (adjusted OR: 2.1 (1.83, 2.42), in contrast to 1.69 (1.56, 1.85) and 1.36 (1.18, 1.55) for individual scores). Figure 3B illustrates the distribution of medication risk scores in PASC cases and controls. We observed that MedRS2 displayed a less normal-like distribution compared to MedRS1, with the presence of two distinct peaks. This characteristic might be attributed to the decreased number of ATC codes included during the acute COVID period, resulting in weaker continuity and stronger category properties in our score. Furthermore, Figure 3B also highlights that the MedRS is more concentrated among PASC cases compared to the separate risk scores, reinforcing its improvement in predicting PASC.

When evaluating the predictive power of the laboratory risk score (LabRS1), as shown in Table 2, we observed that LabRS1 could not discriminate between PASC cases and controls (AAUC = 0.46 (0.41, 0.49)). Additionally, the association between LabRS1 and PASC diagnosis was weak, as indicated by the nonsignificant adjusted odds ratio (adjusted OR = 0.73 (0.45, 1.2), *p*-value = 0.213). These findings suggest that LabRS1 alone may not be sufficient for accurately predicting PASC.

#### 3.3.2. Combined Risk Scores

Building upon the individual contributions of the phenotype risk scores, medication scores, and laboratory risk scores, we aimed to integrate the information captured by these different risk scores. First, we combined PheRS, MedRS, and LabRS1 to create a composite risk score, referred to as AllRS. The evaluation of different risk scores in the same cohort is presented in Table S4. Although AllRS moderately predicted PASC (AAUC(AllRS): 0.64 (0.6, 0.68)), its discrimination power did not demonstrate a substantial improvement compared to the individual risk scores (AAUC(PheRS): 0.64 (0.59, 0.68), Table S4). This lack of improvement was also evident in lower accuracy, reflected by the Brier score (Brier score(AllRS): 0.0963 versus Brier score(PheRS): 0.0955) and a poorer model fit, as suggested by the Pseudo-R^2^ (Pseudo-R^2^(AllRS): 0.071 versus Pseudo-R^2^(PheRS): 0.082). Despite a high OR, a relatively wider confidence interval and higher *p*-value compared with PheRS also do not support an increase in performance. We hypothesize that the limited improvement in predictive power when incorporating LabRS1 may be due to its inherent low predictability, as we have mentioned before. Additionally, the observed correlation between LabRS1 and other risk scores, as shown in Figure S4, consistently exceeded 0.5. This suggests that the information captured by LabRS1 is already encompassed by MedRS, PheRS, and other components in the model. This redundancy in information could also contribute to the lack of improvement when incorporating LabRS1 into the composite risk score.

Thus, recognizing the limited contribution of “LabRS1”, we decided to exclude it from the predictive risk score framework. Instead, we constructed a new composite score called “PheRS&MedRS” by using weighted combinations of only PheRS and MedRS. As presented in Table 2, the model performance of PheRS and MedRS demonstrated further improvement, achieving a Pseudo-R^2^ of 0.094, higher than the individual scores. Despite the absence of a notable increase in PASC discrimination ability (AAUC(PheRS and MedRS): 0.66 (0.63, 0.69) versus AAUC(PheRS): 0.65 (0.62, 0.68)), the higher and more significant adjusted odds ratio (OR[PheRS and MedRS]: 3.68 (3.01, 4.5) versus OR(PheRS): 2.94 (2.48, 3.48), *p*-value (PheRS and MedRS): 2.88 × 10^−37^ versus *p*-value (PheRS): 8.79 × 10^−36^) indicated its stronger risk stratification properties for identifying people at higher risk of PASC. Additionally, Table S4 underscores the superiority of the PheRS and MedRS model, consistently outperforming other predictors across all metrics within the same cohort. This consistency highlights the stability and reliability of our composite risk score in varying sample sizes. Figure 3C also visually demonstrates the increased differentiation in the distribution of PheRS and MedRS between PASC cases and controls, indicating the enhanced discriminatory ability of the combined risk score. Therefore, we adopted the PheRS and MedRS as our final risk score for subsequent analysis and prediction.

### 3.4. PASC Risk Stratification Using a Composite Score

**Figure 4:**
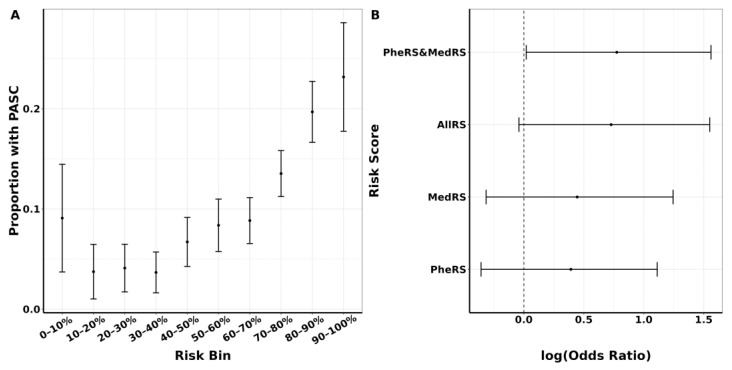
Risk stratification based on aggregate score. (**A**). Distribution of PASC cases by aggregate score (PheRS and MedRS) deciles in the testing data (*n* cases: 454, *n* controls: 3603). (**B**) Odds ratio of PASC corresponding to an RS higher than its 90th percentile in the training set, along with their 95% confidence intervals, for each aggregate score in the testing data (*n* cases: 198, *n* controls: 1553).

In addition to the improved predictive power for PASC, our composite score (PheRS and MedRS) effectively stratified the risk of developing PASC. As depicted in Figure 4A, PheRS and MedRS demonstrated a notable enrichment of PASC cases in the top 10% risk bin (proportion of PASC cases = 23.2%) compared to the lower nine deciles of the score distribution (3.7%–19.7%, Table S5). Particularly, individuals in the top 10% of the PheRS and MedRS exhibited an approximately 2.2-fold enrichment (OR = 2.17 (95% CI: 1.02, 4.76)) in PASC risk compared to those in the remaining 90% of the distribution (Figure 4B), which performed best among all the risk scores. These results indicate that the integration of phenotype risk scores and medication risk scores allows for identifying a large proportion of PASC cases with a 2.2-fold increased risk for PASC in the top 10% compared to the rest. This enrichment is more pronounced if the focus is on only the middle part of the risk distribution; for example, compared to the middle 40–60% of the combined risk distribution, in the top 10%, we see a nearly 13-fold enrichment (OR = 13.14 (95% CI: 6.77, 26.40), Table S5).

## 4. Discussion

In this study, we utilized a cohort of COVID-19-positive individuals from MM, a single medical center, to develop a machine learning-based approach for predicting PASC by integrating multiple health record datasets, including phenotypes, medications, and laboratory biomarkers. Our analysis revealed several important factors in these datasets that are predictive of PASC, including overweight or obesity, use of HMG CoA reductase inhibitors, segmented neutrophils measurement during the pre-COVID period, malaise and fatigue, and use of analgesics or anesthetics during the acute COVID period. When combining the information into individual risk scores based on phenotypes (PheRSs), medications (MedRSs), and laboratory measurements (LabRS1) using SL models, we observed relatively low accuracy in predicting PASC among COVID-19-positive individuals. To overcome this limitation, we developed a combined risk score (PheRS and MedRS), leading to improved predictive power (AAUC(PheRS and MedRS): 0.66 (0.63, 0.69)) in the testing data. Notably, this combined risk score identified 10% of the population with a 2.2-fold increased risk for PASC compared to those in the bottom 90% of its score distribution.

A comparison of our findings with previous studies confirmed many pre-existing health records that might predispose a patient to PASC. For example, according to the SL models on the phenotype data, overweight [23,53], circulatory system diagnoses (e.g., hypertension, complications of heart disease), and respiratory diagnoses (asthma [23,24,54]) were identified as predictive pre-existing conditions for PASC. We also identified newly diagnosed malaise and fatigue [11,18] and respiratory symptoms during the short acute period, including shortness of breath [55], respiratory abnormalities [25], and cough [56,57], to be associated with PASC. Unlike many previous papers, we did not identify mental health factors (e.g., depression and anxiety [58,59]) as highly relevant risk factors, which could be explained by differing definitions of PASC criteria across studies. Additionally, consistent with prior literature, we identified the pre-use of HMG CoA reductase inhibitors and ACE inhibitors as predictors of PASC [60,61]. Moreover, our investigation revealed several laboratory measurements that were associated with PASC diagnoses. For instance, segmented neutrophil counts, known for their correlation with COVID-19 [62], exhibited potential associations with PASC, although limited reports have explored this relationship. However, the constructed pre-COVID laboratory score “LabRS1” demonstrated weak predictive power, both alone and in the composite risk score. One possible explanation for this low performance is the limited number of laboratory measurements and the relatively smaller sample size, which may introduce inaccuracies in the predictive model.

With the establishment of a systematic PASC prediction model, our study not only offers a nuanced understanding of individual risk profiles but also opens avenues for targeted interventions and personalized care strategies, as it bridges existing gaps in the understanding of PASC risk. Specifically, leveraging a comprehensive multi-modal EHR dataset, our model provides a unique opportunity to assess the relative contributions of each data domain to PASC prediction, and assists in informing future studies that may involve primary data collection, enhancing their design and focus. Table S6 summarizes the findings of several recent PASC-prediction-related studies [30,31,35,63,64]. While some of the studies also looked into a large cohort, most of them limited their focus to investigating risk factors from one or two data domains (e.g., phenotypes or medications) and were weaker in assessing the relative contributions of each data domain.

Moreover, our study introduces a novel composite risk score by employing an ensemble learning method. Instead of relying on a single machine learning method, we employed the SL method, which combines random forest, XGBoost, elastic net, and other base algorithms. This approach enabled us to combine the contributions of these models and address the heterogeneity of EHR data features. For comparative purposes, we trained several individual machine learning algorithms, which performed well in previous works (Table S6), on identical cohorts and features, summarized by “PheRS” and “MedRS.” As outlined in Table S7, we contrasted their prediction performance with that of the SL algorithm. The SL algorithm demonstrated superior performance in terms of prediction accuracy, as indicated by the smaller Brier score, suggesting that our SL algorithm is more adaptable to complex data than other individual learners. Furthermore, our constructed pipeline for disease-related risk score construction and prediction offers valuable insights for risk assessment for a wide range of medical conditions beyond PASC.

However, it is important to acknowledge some limitations of our study. First, we performed matching, incl. on age, gender, and race/ethnicity, to adjust for potential confounding and to identify novel predictors (diagnoses, medications, labs, etc.). However, these demographic characteristics seem to be important predictors of PASC [23,65,66]. So, while matching and adjusting for these covariates may have enhanced our ability to identify factors that elevate the risk of PASC, we disregarded these demographic factors as PASC predictors. Future studies are needed to evaluate the combined contributions of these variables in more comprehensive prediction models. Second, the availability of laboratory results was limited, with only 15 laboratory measurements included in our analysis due to a high missing rate. This resulted in lower predictive power for the laboratory measure-based risk scores. Future studies should aim to address this limitation by incorporating a larger set of laboratory measurements [67] or employing imputation methods to improve the predictive accuracy of the models, potentially [68,69,70]. Another limitation pertains to our medication data, which solely includes orders and administrations, but not prescription data. Consequently, the availability of medication data could be skewed towards patients with prior hospitalizations, potentially favoring those who were older or more critically ill. Additionally, although permutation importance served as a robust metric for assessing feature contribution, aligning consistently with SHAP-based results, the inherent randomness in this evaluation warrants consideration. Exploring larger permutation iteration size or alternative importance evaluation methods, such as the LIME (Local Interpretable Model-Agnostic Explanations) method [71,72], could offer valuable insights and enhance the reliability of our feature importance assessments. Furthermore, our study was conducted using data from a single medical center (MM), which may introduce potential biases and limit the generalizability of our findings to other populations or healthcare settings. MM, as an academic medical center with specialized care, may attract certain types of patients. Additionally, attendance at MM is overwhelmingly white and exhibits a higher prevalence of comorbidities. While the analysis is conditional on individuals with confirmed COVID-19, it is crucial to validate the model in other cohorts, particularly those with more diverse patients and those in different healthcare settings (such as outpatient clinics, not specialized academic medical centers).

## 5. Conclusions

PASC pose a significant global public health challenge, impacting millions of individuals. While effective therapies for PASC are still being developed, the use of prediction and risk models can contribute to the reliable identification of individuals at higher risk for PASC and their subcategories, potentially informing preventive and therapeutic efforts. In this study, we aimed to identify pre-existing factors associated with PASC and to develop prediction models for PASC using a comprehensive dataset encompassing phenotype, medication, and laboratory measurements. Through our analysis, we identified several factors predictive of diagnoses of PASC and integrated this information into risk scores that demonstrated moderate predictive capability for PASC. Future studies should further focus on expanding the range of lab measurements included in the analysis, allowing for a more comprehensive assessment of their predictive value for PASC. Furthermore, incorporating additional data sources, such as genetic information, environmental factors, and biomarkers [73,74,75], could provide valuable insights into the underlying mechanisms of PASC and enhance the predictive capability of the models. Ultimately, the development of more accurate and robust prediction models for PASC will have significant clinical implications, enabling early identification of high-risk individuals and facilitating targeted interventions. Such efforts will contribute to the advancement of personalized medicine and the improvement of clinical outcomes for individuals affected by PASC.

## Figures and Tables

**Table 1:** Patient characteristics of COVID-19 patients with (cases) and without observed PASC diagnosis (controls). Case–control matching was based on nearest neighbor matching (age at index date, pre-test years in EHR, post-test years in EHR) and exact matching (gender, primary care at MM, race/ethnicity, quarter of year at COVID-19 index date).

	COVID-19 Patients with PASC Diagnosis	COVID-19 Patients without PASC Diagnosis			
		Unmatched	*p*-Value *	Matched	*p*-Value *
*n*	2287	90,014		22,845	
Age at index date; mean (SD)	47.93 (19.13)	42.29 (22.71)	<0.001	47.23 (19.24)	0.101
Pre-test years in EHR; mean (SD)	11.83 (7.72)	10.48 (7.79)	<0.001	11.74 (7.55)	0.555
Post-test years in EHR; mean (SD)	1.16 (0.70)	0.95 (0.70)	<0.001	1.14 (0.69)	0.337
Female; *n* (%)	1487 (65.0)	51,548 (57.3)	<0.001	14,848 (65.0)	0.999
Primary care at MM; *n* (%)	1328 (58.1)	40,765 (45.3)	<0.001	13,255 (58.0)	0.984
Race/ethnicity; *n* (%)			0.004		0.999
Caucasian/non-Hispanic	1680 (73.5)	64,494 (71.6)		16,800 (73.5)	
African American/non-Hispanic	245 (10.7)	9638 (10.7)		2450 (10.7)	
Other/non-Hispanic or Hispanic	256 (11.2)	9995 (11.1)		2552 (11.2)	
Other/unknown ethnicity	106 (4.6)	5887 (6.5)		1043 (4.6)	
Quarter of year at COVID-19 diagnosis date; *n* (%)			<0.001		1
2020/1	30 (1.3)	668 (0.7)		293 (1.3)	
2020/2	58 (2.5)	1890 (2.1)		567 (2.5)	
2020/3	66 (2.9)	2877 (3.2)		660 (2.9)	
2020/4	289 (12.6)	14,471 (16.1)		2890 (12.7)	
2021/1	259 (11.3)	7967 (8.9)		2590 (11.3)	
2021/2	263 (11.5)	6526 (7.2)		2625 (11.5)	
2021/3	189 (8.3)	4869 (5.4)		1890 (8.3)	
2021/4	331 (14.5)	13,690 (15.2)		3310 (14.5)	
2022/1	319 (13.9)	14,360 (16.0)		3190 (14.0)	
2022/2	187 (8.2)	9214 (10.2)		1870 (8.2)	
2022/3	182 (8.0)	8019 (8.9)		1820 (8.0)	
2022/4	104 (4.5)	5028 (5.6)		1040 (4.6)	
2023/1	10 (0.4)	435 (0.5)		100 (0.4)	
Elixhauser score AHRQ (mean (SD))	4.67 (11.95)	3.75 (10.70)	<0.001	3.94 (11.31)	0.004
BMI (mean (SD))	30.64 (7.88)	29.42 (7.47)	<0.001	30.12 (7.55)	0.003
COVID-19 severity: Severe; *n* (%)	319 (13.9)	5307 (5.9)	<0.001	1358 (5.9)	<0.001
Healthcare worker status: Yes; *n* (%)	63 (2.8)	2527 (2.8)	0.931	802 (3.5)	0.067
Vaccination status *n* (%)			<0.001		0.013
Unvaccinated	1263 (55.2)	50,897 (56.5)		12,565 (55.0)	
After first vaccination	133 (5.8)	3604 (4.0)		1119 (4.9)	
After full vaccination	692 (30.3)	25,288 (28.1)		6751 (29.6)	
After booster	199 (8.7)	10,225 (11.4)		2410 (10.5)	
Population density (%)			0.002		0.248
Quartile 1	572 (25.0)	21,342 (23.7)		5624 (24.6)	
Quartile 2	622 (27.2)	25,533 (28.4)		6697 (29.3)	
Quartile 3	716 (31.3)	26,459 (29.4)		6865 (30.1)	
Quartile 4	197 (8.6)	7592 (8.4)		1830 (8.0)	
Missing	180 (7.9)	9088 (10.1)		1829 (8.0)	
Neighborhood Deprivation Index (%)			0.002		0.274
Quartile 1	832 (36.4)	32,873 (36.5)		8791 (38.5)	
Quartile 2	515 (22.5)	18,746 (20.8)		4871 (21.3)	
Quartile 3	444 (19.4)	16,261 (18.1)		4190 (18.3)	
Quartile 4	316 (13.8)	13,046 (14.5)		3164 (13.8)	
Missing	180 (7.9)	9088 (10.1)		1829 (8.0)	

* *p*-value of differences between COVID-19 patients with a PASC diagnosis and COVID-19 patients without a PASC diagnosis.

**Table 2:** Evaluation of phenotype risk scores (PheRSs) and medication risk scores (MedRSs) on the testing data. All predictors were evaluated while adjusting for covariates. PheRS1: pre-COVID-phenotype risk score; PheRS2: “Acute-COVID New Phenotype Risk Score”; PheRS: combination of PheRS1 and PheRS2; MedRS1: pre-COVID medication risk score; MedRS2: acute COVID new medication risk score; MedRS: combination of MedRS1 and MedRS2; PheRS and MedRS: combination of PheRS and MedRS. AAUC represents the area under the covariate-adjusted receiver operating characteristic of the corresponding predictor. Adjusted OR represents the odds ratio of PASC corresponding to the predictor (centered to the mean and scaled to have a standard deviation of 1) adjusted for the covariates. *p*-Value represents the *p*-value of the corresponding adjusted odds ratio of PASC corresponding to the predictor.

Predictor	Testing Data		AAUC ^a^ (95% CI)	Pseudo-R^2 b^	Brier Score	Adjusted OR ^a^ (95% CI)	*p*-Value
	*n* Cases	*n* Controls					
PheRS1	456	3610	0.56 (0.53, 0.58)	0.033	0.098	1.55 (1.33, 1.82)	4.88× 10^−8^
PheRS2			0.61 (0.58, 0.64)	0.095	0.093	1.94 (1.75, 2.14)	2.97 × 10^−38^
PheRS			0.65 (0.62, 0.67)	0.093	0.094	2.96 (2.49, 3.5)	6.12 × 10^−36^
MedRS1	525	5436	0.53 (0.5, 0.56)	0.025	0.079	1.36 (1.18, 1.55)	1.16 × 10^−5^
MedRS2			0.6 (0.58, 0.63)	0.068	0.077	1.69 (1.56, 1.85)	1.30 × 10^−33^
MedRS			0.61 (0.58, 0.64)	0.057	0.078	2.1 (1.83, 2.42)	1.76× 10^−25^
LabRS1	209	1895	0.46 (0.41, 0.49)	0.025	0.088	0.73 (0.45, 1.2)	0.213
PheRS	454	3603	0.65 (0.62, 0.68)	0.089	0.094	2.94 (2.48, 3.48)	8.79 × 10^−36^
MedRS			0.59 (0.56, 0.62)	0.046	0.097	1.99 (1.68, 2.35)	1.04 × 10^−15^
PheRS and MedRS			0.66 (0.63, 0.69)	0.094	0.094	3.68 (3.01, 4.5)	2.88 × 10^−37^

^a^ Adjusted for age at index date, gender, race/ethnicity, BMI, Elixhauser score, population density, NDI, healthcare worker status, vaccination status, pre-test years in EHR, and COVID-19 severity. ^b^ Nagelkerke (Cragg and Uhler).

## Data Availability

Data cannot be shared publicly due to patient confidentiality. The data underlying the results presented in the study are available from the University of Michigan Precision Health Analytics Platform at https://precisionhealth.umich.edu/tools-resources/data-access-tools/ accessed on 28 September 2023 for researchers who meet the criteria for access to confidential data.

## Supplementary Materials

The following supporting information can be downloaded at: https://www.mdpi.com/article/10.3390/jcm12237313/s1, Table S1: List of Clinical laboratory measurements and the results of univariate regressions. Table S2: Feature Importance for covariates on the training set. Table S3: Weights for combining risk scores. Table S4: Evaluation of Phenotype Risk Scores (PheRS) and Medication Risk Scores (MedRS) on the testing data. Table S5: Evaluation of risk stratification using PheRS&MedRS on the testing data. Table S6: Comparative analysis of Super Learner and individual base learners on the identical testing cohort. Table S7: Summary of findings from PASC-prediction related literatures. Figure S1: Study Design Schematic. The time periods in this study were defined relative to day 0, which corresponds to the date of first diagnosis or testing as COVID-19 positive (index date). Figure S2: SHAP-based Feature Importance Plots for Phenotype and Medication Data on the Training set. Figure S3: Feature importance plots for lab results on the training set. Figure S4: Correlation plot of five risk scores on the testing set (*n* cases: 451, *n* controls: 1553). References [30,31,35,68,69] are cited in the Supplementary Materials.
