# Supplementary material for: Using Multi-Modal Electronic Health Record Data for the Development and Validation of Risk Prediction Models for Long COVID Using the Super Learner Algorithm
Source: J Clin Med. 2023 Nov 25;12(23):7313. doi: 10.3390/jcm12237313 (PMC10707399; doi:10.3390/jcm12237313)
Supplement: Supplementary file 1 [file jcm-12-07313-s001.zip › jcm-2665924-supplementary.pdf]

Supplementary Material for: Using multi-modal EHR data for Development and Validation of Risk Prediction Models for Long COVID using the Super Learner Algorithm

Supplementary Results

Table S1. List of Clinical laboratory measurements and the results of univariate regressions. The "Clinical Lab (units)" column indicates the name of the lab measurement used in our study and its corresponding unit. The "Odds Ratio" column represents the odds ratio (OR) of the lab measurement estimated from Equation 1. The "95% CI LB" and "95% CI UB" columns indicate the lower and upper bounds of the estimated odds ratio, respectively. The "Complete Cases" column displays the number of observations containing the corresponding measurement in the training set.

**Clinical Lab (units)**	**Odds Ratio**	**95% CI LB**	**95% CI UB**	**p-value**	**Complete Cases**
Hematocrit (%)	1.15	1.06	1.24	0.001	10,108
Hemoglobin (g/dL)	1.14	1.06	1.24	0.001	10,086
Red blood cell count (M/uL)	1.13	1.04	1.21	0.002	10,086
Segmented neutrophils (%)	0.92	0.85	0.99	0.020	8,461
Basophils (%)	1.08	1.00	1.16	0.039	8,460
Absolute Neutrophil Count (K/uL)	0.92	0.85	1.00	0.052	8,441
Red cell distribution width (%)	0.93	0.86	1.00	0.055	10,085
Immature Granulocyte %	0.92	0.84	1.01	0.081	8,428
Calcium level (mg/dL)	1.06	0.99	1.14	0.091	10,172
White blood cell count (K/uL)	0.93	0.84	1.02	0.132	10,086
Alkaline phosphatase (IU/L)	0.94	0.87	1.02	0.165	9,083
Anion Gap (mmol/L)	1.05	0.98	1.13	0.184	9,595
Absolute Early Gran Count (K/uL)	0.92	0.82	1.05	0.210	8,440
Sodium level (mmol/L)	1.04	0.97	1.12	0.260	10,169
Cholesterol (mg/dL)	0.95	0.88	1.04	0.277	7,119
pH of body fluid (N/A)	1.05	0.96	1.15	0.295	5,681
Low density lipoprotein cholesterol (mg/dL)	0.96	0.88	1.04	0.309	7,017
Protein level (g/dL)	1.04	0.96	1.12	0.353	9,111
Creatinine level (mg/dL)	1.03	0.97	1.09	0.371	10,286
Specific gravity (urine) (N/A)	0.97	0.89	1.06	0.516	5,680
Absolute Basophil Count (K/uL)	1.02	0.95	1.10	0.539	8,440
Hemoglobin a1c (%)	1.03	0.95	1.11	0.543	6,805
Alanine transaminase (IU/L)	0.98	0.89	1.07	0.587	8,968
Platelet count (K/uL)	1.02	0.95	1.09	0.627	10,085
Mean corpuscular hemoglobin concentration (g/dL)	1.02	0.95	1.10	0.627	10,086
Potassium level (mmol/L)	0.98	0.92	1.06	0.644	10,170
Absolute Eosinophil Count (K/uL)	0.98	0.91	1.06	0.688	8,440

Eosinophils (%)	0.99	0.92	1.06	0.729	8,460
Carbon dioxide (CO2) (mmol/L)	1.01	0.94	1.09	0.749	10,165
Mean corpuscular hemoglobin (pg)	1.01	0.94	1.09	0.778	10,086
Thyroid-stimulated hormone (mIU/L)	1.01	0.94	1.09	0.780	7,330
Glucose level (mg/dL)	0.99	0.93	1.06	0.800	10,273
Bilirubin, total (mg/dL)	0.99	0.92	1.07	0.814	9,118
Chloride (mmol/L)	0.99	0.93	1.06	0.820	10,169
Triglycerides (mg/dL)	0.99	0.91	1.08	0.824	7,168
Urea nitrogen (mg/dL)	1.01	0.94	1.08	0.830	10,170
Mean corpuscular volume (fl)	1.01	0.94	1.08	0.851	10,086
Mean platelet volume (fl)	1.00	0.93	1.07	0.940	10,066
Absolute Monocyte Count (K/uL)	1.00	0.93	1.07	0.954	8,441
Monocyte %, coulter (%)	1.00	0.93	1.07	0.954	8,461
Absolute Lymphocyte Count (K/uL)	1.00	0.94	1.07	0.983	8,441
Lymphocyte % (coulter) (%)	1.00	0.94	1.07	0.983	8,461

Table S2: Feature Importance for covariates on the training set. All importance has been multiplied by 100. Importance was evaluated by the permutation importance (defined as the decrease of model AUC after the corresponding feature was randomly shuffled) based on the superlearner model with the covariates being adjusted. Each importance was estimated by 10 Monte Carlo replications. Models represent the data included in the superlearner model.

Covariates	Model					
	Covariates Only	Pre COVID-Phenotype+ Covariates;	Post COVID New Phenotype +Covariates	Pre COVID-Medication + Covariates;	Post COVID New Phenotype +Covariates	Pre COVID-Lab Results+ Covariates;
Age at index date	13.2	0.7	5.7	3.1	5.8	3.2
BMI	12.5	0.9	4.8	3.6	5.7	3.0
COVID 19 Severity	2.9	1.1	0.2	2.0	0.2	5.7
Elixhauser Comorbidity Score	8.1	0.6	2.6	0.4	1.5	2.8
Gender	2.0	0.1	0.9	0.8	1.6	0.4
Health Care Worker Status	0.3	0.0	0.1	1.2	1.8	0.1
Neighborhood Deprivation Index (quartiles)	3.7	0.3	1.7	0.1	0.3	1.4
Population Density (quartiles)	2.6	0.2	1.6	1.0	1.9	1.2
Pre-test years in EHR	10.8	0.8	3.8	1.8	3.3	3.0
Race: African American / Non-Hispanic	0.5	0.0	0.2	0.2	0.5	0.2
Race: Caucasian / Non-Hispanic	0.8	0.1	0.6	0.3	0.6	0.3
Race: Other / Non-Hispanic or Hispanic	0.4	0.0	0.3	0.2	0.4	0.3

Race: Other / Unknown Ethnicity	0.2	0.0	0.1	0.1	0.2	0.1
Vaccination Status	3.5	0.4	1.5	2.6	4.8	2.2

Table S3 Weights for combining risk scores. "Combined RS" represents the risk scores generated by combining distinct risk scores. "Predictor" represents the distinct risk scores used for combining. "Weights" represents the adjusted "weight" (estimated coefficient) of corresponding risk score. Each predictor was standardized (mean = 0 and sd = 1) before calculating the weights and before calculating the combined risk scores.

**Combined RS**	**Predictor**	**Weight***
PheRS	PheRS1	0.62111
	PheRS2	0.37889
MedRS	MedRS1	0.65624
	MedRS2	0.34376
AllRS	PheRS	0.38457
	MedRS	0.25626
	LabRS1	0.35917
PheRS&MedRS	PheRS	0.60317
	MedRS	0.39683

** Weights have been standardized to sum to 1.*

Table S4: Evaluation of Phenotype Risk Scores (PheRS) and Medication Risk Scores (MedRS) on the testing data. All predictors were evaluated while adjusting for covariates. PheRS1: pre-COVID-19 Phenotype Risk Score; PheRS2: "acute-COVID-19 New Phenotype Risk Score; PheRS: Combination of PheRS1 and PheRS2; MedRS1: pre-COVID-19 Medication Risk Score; MedRS2: acute-COVID-19 New Medication Risk Score; MedRS: Combination of MedRS1 and MedRS2; LabRS1: pre-COVID-19 Lab Risk Score; PheRS&MedRS: Combination of PheRS and MedRS. AllRS: Combination of PheRS, MedRS and LabRS1. AAUC represents the covariate-adjusted receiver operating characteristic of the corresponding predictor. OR represents the odds ratio of PASC corresponding to a predictor higher than its 75th percentile (as determined by the the training set) on the testing set. Adjusted OR represents the odds ratio of PASC corresponding to the predictor (centered to the mean and scaled to have a standard deviation of 1) adjusted for the covariates. P-value represents the p-value of the corresponding adjusted odds ratio of PASC corresponding to the predictor.

**Predictor**	**Testing Data**		**AAUC[a] (95% CI)**	**Pseudo-R^2 [b]**	**Brier Score**	**Adjusted OR[a] (95% CI)**	**P-value**
	**n cases**	**n controls**					

PheRS1	198	1553	0.55 (0.51, 0.59)	0.033	0.0987	1.55 (1.22,1.96)	3.3E-04
PheRS2			0.61 (0.56, 0.65)	0.081	0.0949	1.77 (1.53,2.05)	9.5E-15
PheRS			0.64 (0.59, 0.68)	0.082	0.0955	2.73 (2.11,3.54)	3.5E-14
MedRS1			0.50 (0.45, 0.54)	0.019	0.0993	1.07 (0.82,1.39)	0.63
MedRS2			0.62 (0.57, 0.66)	0.071	0.096	1.75 (1.5, 2.05)	3.1E-12
MedRS			0.59 (0.55, 0.64)	0.042	0.098	1.88 (1.44,2.47)	4.1E-06
LabRS1			0.46 (0.42, 0.5)	0.02	0.0992	0.76 (0.45,1.3)	0.32
PheRS&MedRS			0.66 (0.61, 0.7)	0.087	0.0955	3.6 (2.61, 4.96)	6.6E-15
AllRS			0.64 (0.6, 0.68)	0.071	0.0963	5.04 (3.18,8.00)	6.6E-12

[a] Adjusted for age at index date, gender, race/ethnicity, BMI, Elixhauser Score, population density, NDI, health care worker status, vaccination status, pre-test years in EHR, and COVID-19 severity

[b] Nagelkerke [Cragg and Uhler])

Table S5: Evaluation of risk stratification using PheRS&MedRS on the testing data. Risk Bin represents the ten deciles of PheRS&MedRS based on the training set. Proportion indicates the proportion of PASC diagnoses in the corresponding risk bin in the testing set. OR (95% CI) indicates the estimated odds ratio (and its 95% CI) of PASC corresponding to each risk decile.

Risk Bin	Proportion (95%CI)	OR (95% CI)
0-10%	0.091 (0.037,0.14)	1.15 (0.54,2.24)
10-20%	0.037 (0.01,0.065)	0.45 (0.19,0.95)
20-30%	0.041 (0.017,0.065)	0.50 (0.25,0.93)
30-40%	0.037 (0.016,0.057)	0.46 (0.23,0.84)
40-50%	0.067 (0.043,0.092)	-
50-60%	0.084 (0.058,0.11)	
60-70%	0.088 (0.065,0.11)	1.18 (0.80,1.74)
70-80%	0.14 (0.11,0.16)	1.98 (1.43,2.75)
80-90%	0.2 (0.17,0.23)	4.04 (2.89,5.68)
90-100%	0.23 (0.18,0.29)	13.14 (6.77,26.40)

Table S6: Comparative analysis of Super Learner and individual base learners on the identical testing cohort (for “pre-COVID-19 phenome” and “acute-COVID-19 new phenome”: 454 cases and 3610 controls; for “pre-COVID-19 medicome” and “acute-COVID-19 new medicome”: 525 cases and 5436 controls). The models were trained on the same training cohorts and same features. “Data Domain” represents the individual data domain used for model training. “Learner“ represents the name of the learner used for model training.

Data Domain	Learner	Brier Score
pre-COVID-19 phenome	Super Learner	0.0780
	Random Forest	0.0790

	GLM	0.0998
	Elastic Net	0.0785
	XGBoost	0.0819
acute-COVID-19 new phenome	Super Learner	0.0945
	Random Forest	0.0972
	GLM	0.108
	Elastic Net	0.0960
	XGBoost	0.0997
pre COVID-19 medicome	Super Learner	0.0787
	Random Forest	0.0814
	GLM	0.0829
	Elastic Net	0.0798
	XGBoost	0.0843
acute-COVID-19 new medicome	Super Learner	0.0780
	Random Forest	0.0806
	GLM	0.0808
	Elastic Net	0.0791
	XGBoost	0.0812

Table S7: Summary of findings from PASC-prediction related literatures. "Main Field of Predictors" represents the primary data domain used for prediction (excluding demographic features.)

Publication	Prediciton Method	Cohort Size	Evaluation Metric	Main Field of Predictors	Major findings on risk factors
Surdre et al. (2021) [31]	Random Forest	4,182	AUC: 0.76	Phenotype	Fatigue, headache, dyspnea, hoarse voice and myalgia
Pfaff et al. (2022) [30]	XGBoost	97,995	AUC: 0.92	Phenotype, Medication	Rate of health-care utilisation, patient age, dyspnoea, and other diagnosis and medication information
Binka et al. (2022) [68]	Elastic Net	168,111	AUC: 0.93	Phenotype	Shortness of breath/dyspnea and malaise and fatigue
Fritsche et al. (2023) [35]	Logistic Regression	63,675	AAUC: 0.615	Phenotype	Irritable bowel syndrome, concussion, nausea/vomiting, predominantly respiratory symptoms, and circulatory symptoms
Kessler et al. (2023) [69]	Light Gradient Boosting Machine	272,588	AUC: 0.84	Phenotype	Somatoform disorders, migraine, back pain, asthma, malaise and fatigue

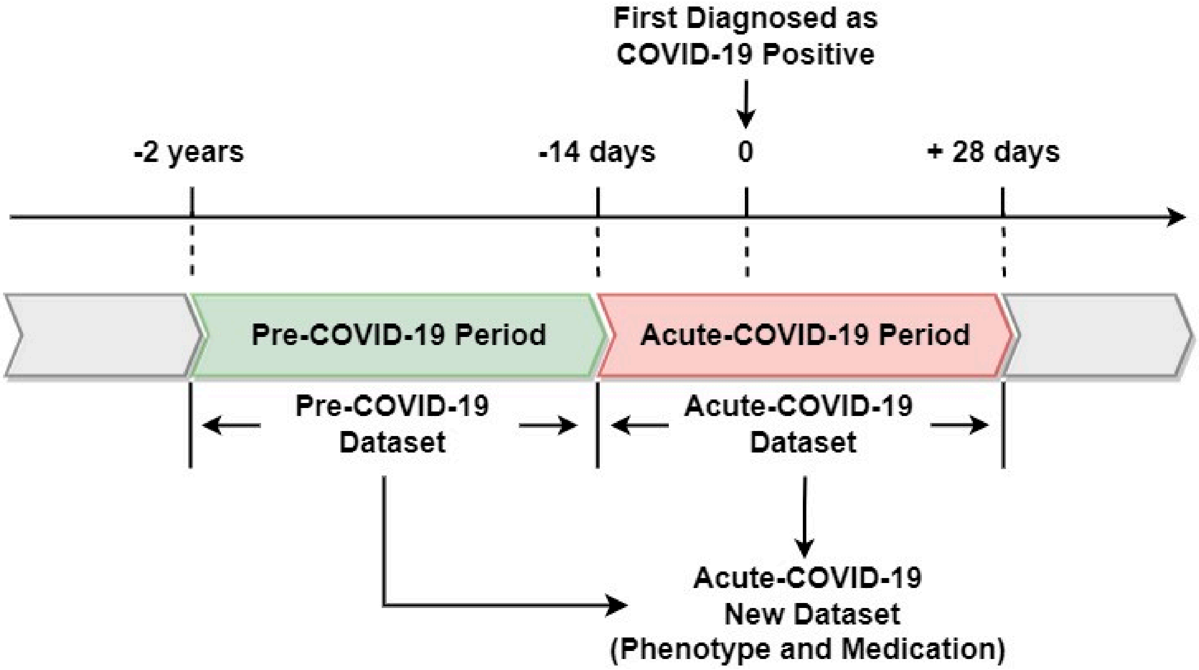

Figure S1: Study Design Schematic. The time periods in this study were defined relative to day 0, which corresponds to the date of first diagnosis or testing as COVID-19 positive (index date). The pre-COVID-19 period spanned from -2 years to -14 days; the acute-COVID-19 period covered the timeframe from -14 days to +28 days. In addition, the acute-COVID-19 new dataset was generated to include newly diagnosed conditions or medications that exclusively appeared during the acute-COVID-19 period.

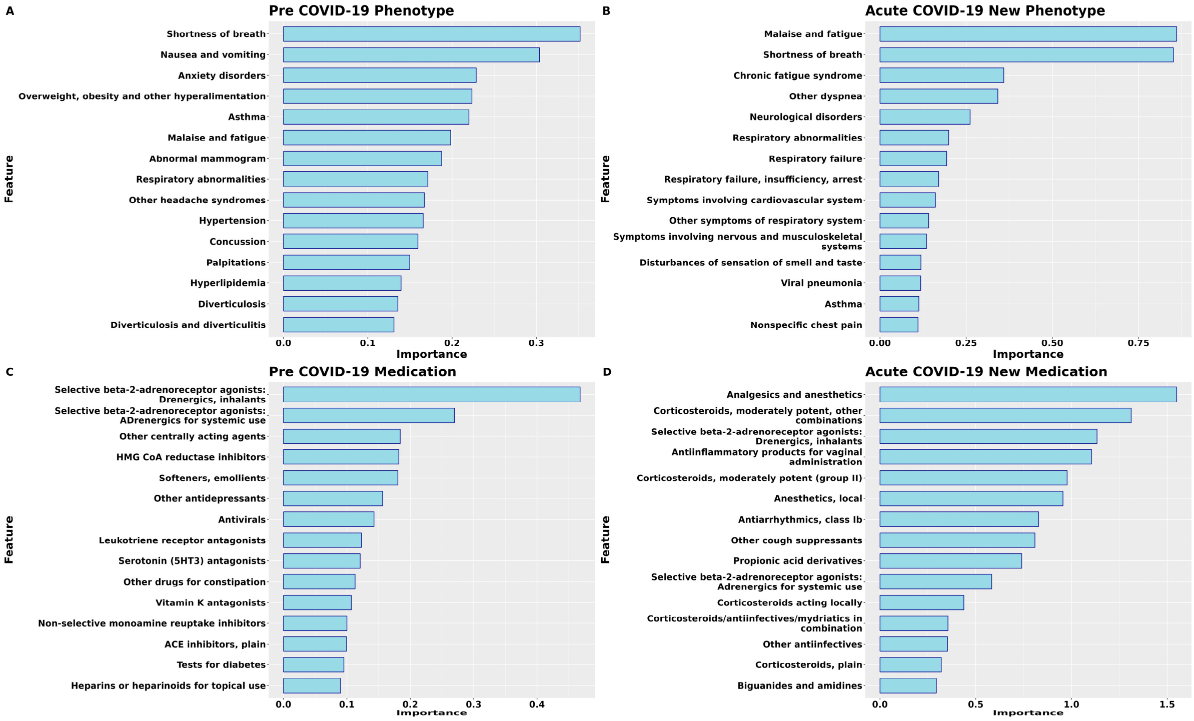

Figure S2: SHAP-based Feature Importance Plots for Phenotype and Medication Data on the Training set. All importance has been multiplied by 100. Importance was evaluated by the Shapley method based on the Super Learner model with the covariates being adjusted. Only 15 features with highest importance were presented. A: Feature Importance Plot for phenotypes in the Super Learner (SL) model with pre-COVID phenotypes; B: Feature Importance Plot for phenotypes in the SL model with acute-COVID new phenotypes; C: Feature Importance Plot for medications in the SL model with pre-COVID medications; D: Feature Importance Plot for medications in the SL model with Acute-COVID new medications.

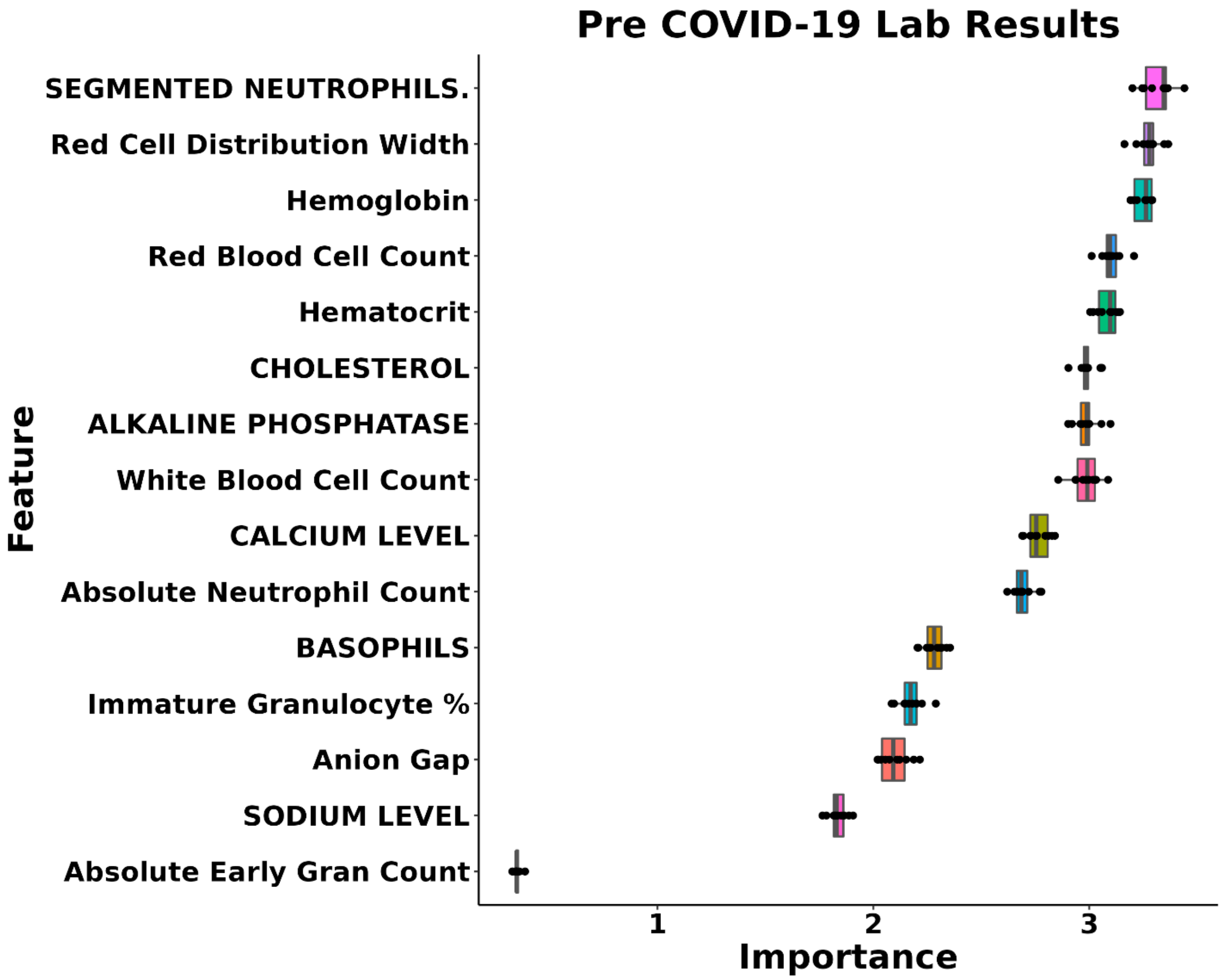

Figure S3: Feature importance plots for lab results on the training set. All importance has been multiplied by 100. Importance was evaluated by the permutation importance (defined as the decrease of model AUC after the corresponding feature was randomly shuffled) based on the super learner model with the covariates being adjusted. Each importance was estimated by 10 Monte Carlo replications.

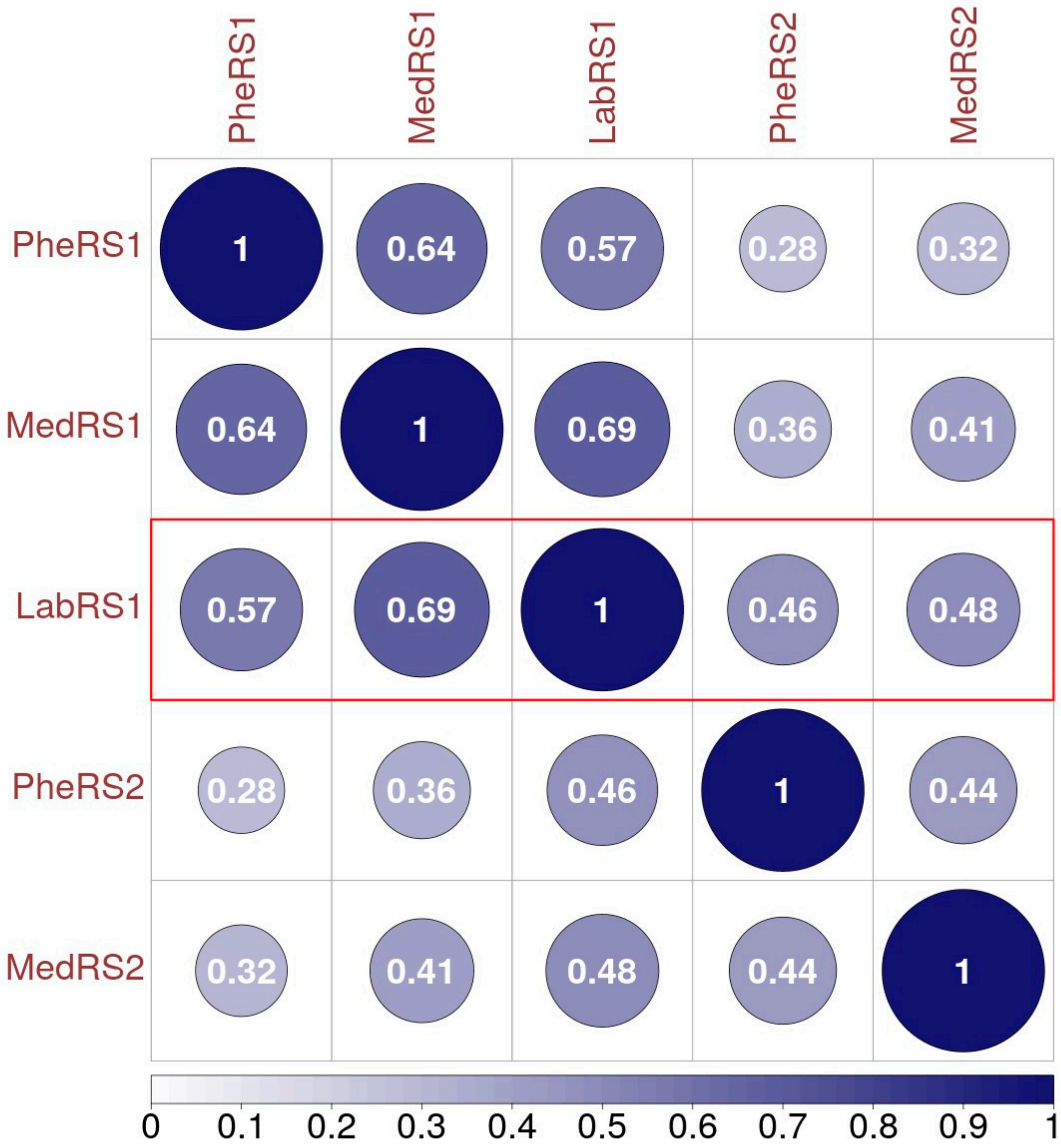

Figure S4: Correlation plot of five risk scores on the testing set (n cases: 451, n controls: 1553). The numeric values illustrate the Pearson correlation coefficients between the five risk scores, namely pre-COVID-19 Phenotype Risk Score (PheRS1), Pre-COVID-19 Medication Risk Score (MedRS1), Acute COVID-19 new Phenotype Risk Score 2 (PheRS2), Acute COVID-19 new Medication Risk Score 2 (MedRS2), and Pre-COVID-19 Laboratory Risk Score (LabRS1). The red frame highlights the correlation between LabRS1 and the other scores.
